# Depression and alexithymia on weight perception in patients with metabolic syndrome and type 2 diabetes

**DOI:** 10.1186/s13098-017-0222-4

**Published:** 2017-05-12

**Authors:** Maria Cristina de Oliveira Regina, Marcos Antonio Tambascia

**Affiliations:** 1 Department of Human Being Development and Rehabilitation, Medical Faculty of State University of Campinas, Campinas, Brazil; 2 Department of Internal Medicine, Endocrinology Metabolic Syndrome and Diabetes, Medical Faculty of State University of Campinas, Campinas, Brazil

**Keywords:** Perception of weight, Actual body mass index, Reported or declared body mass index, Dyslipidemia, Hypertension, Type 2 diabetes mellitus, Alexithymia, Depression, Anxiety and psychological aspects

## Abstract

**Background:**

Obesity’s increasing follows decreased perception of weight status in obese persons, mainly female, undergoing age-related changes.

**Objective:**

To study weight perception and psychological alterations associated to MS and T2DM.

**Methods:**

200 patients selected from Metabolic Syndrome Outpatient Clinic of University of Campinas. Instruments: Beck Depression and Beck Anxiety Inventories’, Toronto Alexithymia Scale-26s, questionnaire and data from reports. Approved by Unicamp Research Ethic Committee.

**Results:**

Patients aged 18–40 years perceived their weight higher than actual (A < D) (p = 0.0272), amongst untreated hypertensive (p = 0.037). ≥41 years old patient’s subdivided into A = D and A > D. A = D had 4.3 more chances to be alexithymic than A < D. 35% of A < D accepted their physical appearance, contrarily A = D (66%) and A > D (69%) (p = 0.0018). 50% of A < D felt offended by social aggression due to their weight; A = D (20%) and A > D (34%) (p = 0.007). 3.6 more chances of A > D than A < D using anti-hypertensive drugs (p = 0.021) (≥41 years old) and 3.5 more chances to perceive A = D (41–60 years old) (p = 0.023). A = D presented 3.8 more chances of depression than A < D and 4.3 more chances of alexithymia than A < D (62% of 41–60 year-old patients with higher cholesterol, mainly LDL and hyper-triglycerides). A = D with alexithymia, partially linked with higher cholesterol, suggests neuroinflammation due to hypertriglycerides. Females, who declared had been anteriorly made diet as treatment to lose weight were exactly those who perceived their weight A > D (45%, p = 0.0091).

**Conclusions:**

Age as a period of development, in which cultural influences occurs, was a factor in weight misperception. A < D and A > D were distinct in age, history of obesity and BMI.

## Background

Evidence supports that increasing prevalence of obesity has been accompanied by decreasing perception of weight status in obese people [1]. Female were likelier than male, to consider themselves as overweight across each BMI category, and more attempted to lose weight. Both males and females with higher BMI were associated with lower chance of trying to lose weight than patients slightly overweight [1]. Process of aging can affect female body image perception. Female with body image dissatisfaction, or who perceived themselves as “unattractive” had higher inaccuracy regarding perception of their bodies [2]. Middle-aged women with poor body image are more likely to have clinically significant levels of symptoms of depression [2]. As obesity has increased in the United States [3], studies have found that Americans’ perception of their own weight has not often aligned with their actual BMI. The accuracy of weight perception was associated with gender, race, and education level in a study regarding dissatisfaction among adolescents in 24 countries [4]. Body weight dissatisfaction was highly prevalent and more common in girls than boys were, if overweight than non-overweight adolescents and if older than young.

The number of type 2 diabetes mellitus (T2DM) is increasing in every country, especially in those with low and middle-income. T2DM has often found between ages 40–59 [5].

Metabolic syndrome (MetS) is a cluster of cardiometabolic risk factors that includes obesity, insulin resistance, dyslipidemia and hypertension [6, 7]. MetS has been considered a pre-diabetes state [8]. It can be caused by genetic associations [9] and other factors as obesity, medications for anxiety, depression, psychoses, HIV, allergies, respiratory diseases and frequent exposure to high levels of cortisol in prolonged stress [10]. Visceral obesity is a relevant factor [10]. The MetS has been recognized by the WHO and other important institutions such as: The National Cholesterol Education Program (NCEP), and The Adults Treatment Panel III (APTIII) [11].

Obesity, as important factor in MetS, increases the incidence to T2DM. Other factors as family history of diabetes, unhealthy or obesogenic diet, physical inactivity, increasing age, high blood pressure, ethnicity, impaired glucose tolerance, history of gestational diabetes and poor nutrition during pregnancy [5] can contribute to cause MetS.

A decline in complications has been associated with T2DM, but not in rates of amputation, stroke or end-stage of renal disease [12]. People who developed T2DM had presented increased glucose values and reduced insulin sensitivity 13 years before the diagnosis [8].

Aging is associated with increasing of total body weight and visceral adiposity [12]. Despite BMI continues stable, percentage of fat tissue is higher with age [13] and visceral adiposity linked to MetS. Prevention and weight control, in MetS patients, mainly in patients with T2DM, is very important due to the damages caused by the continuous state of pro-inflammation [6]. Overweight perception is a source to conduct patients to control those damages.

Alexithymia is the difficulty in identifying and describing feelings [14], an alteration on emotional regulation [15]. It represents a deficit in cognitive processing of affection, which predisposes to several physical and psychiatric illnesses. Neurologically, the physiological motor expressive area is active, more than cognitive experiential domain of the system of emotional response [16]. It has especially been linked with psychiatric and psychosomatic disorders [16] such as somatization [14], depression and anxiety [17–21]. Alexithymia is elevated in people with both eating and drug-related disorders [22]. They have link with compulsive behavior disorders. It is described as the difficulty of identifying feelings in other people too, including main basic feelings [17, 23–32]. Studies regarding alexithymia have shown that patients with alexithymia present hypersensitivity to bodily sensations due to neural features, which are activated more on physiologic or motor expressive levels than on cognitive-domains of the emotional response system [32].

Depression-related biological alterations in the hypothalamic–pituitary–adrenal cortex axis, occurs due to subclinical inflammation or as resultant of the sympathetic nervous system, were not relevantly linked to increased risk of diabetes [33–36]. A modestly bidirectional association between T2DM and depression [37] was found in a meta-analyses of cohort studies. Other studies showed no conclusive bidirectional influence between them [38].

Some studies pointed out the association of depression with pro-inflammatory markers or inflammatory states, demonstrating a link to immunological activity. Some of those inflammatory markers are the same found in obese states [35, 36].

Diabetes, as well as other chronic disorders, increases the risk of depression in equal dimension. The possibility of a faster cognitive decline due to T2DM exists as long as related to duration of the disease and to the glycemic control [37]. It was observed [39] that the brain, under higher BMI, is related to Alzheimer’s disease [39].

Few studies have been conducted regarding perception of obesity, MetS and alexithymia, mainly focusing on depression and anxiety, and showing types of weight perception. Probably because of pro-inflammatory state, it is possible that obesity has a link with alexithymia. Alteration of perception in general can be affected by this factor. The eventual relationship between MetS and Alexithymia may probably be demonstrated. Certain health parameters that compound MetS can have association with both alexithymia and weight perception alterations. If the weight perception is affected, it is possible to establish tendencies considering the levels of obesity; and to establish the levels of alexithymia, mainly if alexithymia is, secondarily, linked to depression and/or anxiety. Demographic items, such as age and gender may also affect those associations.

## Methods

Sample: 200 patients was, randomly, selected from 800 patients attended per year, in Metabolic Syndrome Outpatient Clinic of the State University of Campinas (Brazil); they accepted to participate in the study using the Free and Informed Consented Form. Twenty of them were excluded because they did not adequately complete one or more of the tests or the questionnaire; 10 were also excluded due to diseases which could be confounding factors. The Research Ethics Committee of the University of Campinas approved the study. The participants ranged from 18 to 71 years, both genders. It is a cross-sectional study. Instruments: a questionnaire with closed questions (socio-demographic items, attitudes and psychological aspects related to obesity), Beck Depression Inventory, Beck Anxiety Inventory and Toronto Alexithymia Scale-26 and data from reports concerning control of metabolic disorder. Diagnosis of type 2 diabetes mellitus was considered if fasting plasma glucose was higher than 126 mg/dl; or if there was use of medication for them. Classification of obesity was based on body mass index that is calculated by the formula: weight/height 2 and considered as follows: between 25 and 30 as overweight, 30 and 35 as obesity grade I, 35 and 40 as obesity grade II and more than 40 as severe obesity or grade III. Statistics: Fisher test to study the prevalence and Chi Square for correlations among variables and study of prevalence; Multiple Linear Regression and Multiple Correspondence Factor Analyses in order to analyze co-variance and the chance that they happen together.

## Results

### Demographic aspects

Weight perception presented a differential between 18 and 40 years old patients: actual BMI > declared BMI (A > D) (18%) and Actual BMI < declared BMI (A < D) (39%). Married was prevalent amongst patients who perceived their weight A > D; A < D amongst unmarried was prevalent among A < D patients (see Table 1). Formal education was not significant in all types of weight perception.

**Table 1:** Differences between actual body mass index (A) − declared body mass index (D)

	A = D	A > D	A < D	Total	p value
Gender					0.0874
Female	39 (74%)	36 (65%)	39 (85%)	114 (74%)	
Male	14 (26%)	19 (35%)	7 (15%)	40 (26%)	
Total	53 (34%)	55 (36%)	46 (30%)	154 (100%)	
Civil state					0.0657
Married	30 (57%)	40 (73%)	23 (51%)	93 (61%)	
Not married	23 (43%)	15 (27%)	22 (49%)	60 (39%)	
Total	53 (35%)	55 (36%)	45 (*29%*)	153 (100%)	
Age intervals					*0.0272*
18–40 years old	12 (23%)	10 (18%)	*18 (39%)*	40 (26. %)	
41–60 years old	32 (62%)	34 (62%)	27 (59%)	93 (61%)	
≥61 years old	8 (16%)	11 (*20%*)	1 (2%)	20 (13%)	
Total	52 (34%)	55 (*36%*)	*46 (30%)*	153 (100%)	
Anti-hypertensive					*0.0372*
No	37 (70%)	39 (69.6%)	*41 (89%)*	117 (75%)	
Yes	16 (30%)	17 (30%)	*5 (11%)*	38 (24.5%)	
A > D × A < D (with anti-hypertensive) A = D	odds ratio = 3.574 odds ratio = 3.546		p = 0.0219 p = 0.0239		
Anti-depression					0.3246
No	47 (89%)	47 (84%)	43 (93%)	137 (88%)	
Yes	6 (11%)	9 (16%)	3 (7%)	18 (12%)	
Body mass index					
I	6 (21.4%)	0 (0.0%)	22 (78.6%)	28 (100%)	*<0.0001*
II	28 (41.2%)	16 (23.5%)	24 (35.3%)	68 (100%)	
III	0 (0.0%)	*40 (67.8%)*	19 (32.2%)	59 (100%)	
Beck depression inventory					*0.0499*
Low level	33 (70%)	36 (71%)	36 (90%)	105 (76%)	
Moderate/severe	14 (30%)	15 (29%)	4 (10%)	33 (24%)	
Total	47	51	40	138 (100%)	
Moderate or severe depression	A > D × A < D A = D × A < D	p = 0.0337 p = 0.0297		odds ratio = 3.648 odds ratio = 3.817	
Toronto Alexithymia Scale-26					*0.0161* (Fisher)
No	1 (2%)	2 (4%)	6 (16%)	9 (7%)	
Probable alexithymia	8 (17%)	13 (25%)	13(34%)	34 (25%)	
Alexithymia	39 (81%)	37 (71%)	19 (50%)	95 (69%)	
Total	48	52	38	138 (100%)	
Alexithymia/BMI actual × BMI declared	A > D × A < D A = D × A < D	p = 0.0368 p = 0.0029	odds ratio = 2.533 odds ratio = 4.333		

^a^Anxiety measured by Beck Anxiety Inventory did not present statical significance according to Chi square test: p = 0.1497; it was observed only the following difference, comparing on their actual BMI and their declared BMI (A < D): minimal level of anxiety 68% and moderate or high level of anxiety 32%

^b^Prevalence of probable alexithymia (38%) was among patients who did not use anti-hypertensive medication and did not have controlled hypertension. Prevalence (86%) of alexithymia among patients with controlled hypertension and used anti-hypertensive medication (p = 0.069)

In male gender there was particularities to incorrectly perceive weight (BMI): A > D = 35% and A < D = 15%. In A < D, females were prevalent: fewer male patients perceiving their weight A < D. According to multiple correspondence analyses test, male patients presented certain features (55%): they did not make any treatments for weight loss, were ≥BMI III; they disapproved their own physical appearance (possibly they were not able to transform their non-acceptance into actions to ameliorate appearance), did not feel upset due to their disease (denying mechanism), diet or medical treatment. They also declared not having difficulty to expose their sentiments, however had alexithymia and severe or moderate depression and anxiety.

### Hypertension, dyslipidemia and weight perception

Hypertension could result of (or from) hyperinsulinemia induced by obesity and/or T2DM and other factors. Systolic pressure were different between patients aged ≤40 years old (higher) and 41–60 years old (lower); and ≥61 years old (higher) (0.026). Literature evaluates differently the ideal level of systolic blood pressure, considering age. [39, 40]. Higher diastolic pressure was in aged ≤40 years old. Hypertension, prevalent in ≤40 years old, possibly was initial effect of lesser sensibility of insulin and inadequate eating habits; in patients aged ≥61 years old, probably was result of a longer history of obesity and worse management of insulin prescribed.

A < D was present in hypertension without use of anti-hypertensive, in patients with ≤40 years old; while use of anti-hypertensive was linked with A > D or A = D. It means that anti-hypertensive did not influence weight perceptions in A = D. Some factor associated to hypertension can be linked with A > D.

Patients who were not making use of anti-hypertensive drugs and perceived their weight A < D were statistically significant (see Table 1). Patients using anti-hypertensive presented 3.6 more chances of A > D than A < D or 3.5 more chances to perceive A = D than A < D. It was not studied how frequently and correctly anti-hypertensive drug were used by them or how accessible for patients it was. In A = D the use of anti-hypertensive did not affect weight perception. Then A > D could be more affected by anti-hypertensive probably due to wrong way to use it, even though correct prescription and orientation.

In patients who perceived A > D: 46% were not previously submitted to diet to lose weight (p = 0.0091), which is in agreement with literature. It was observed in male gender in multiple correspondence analysis test. The lack of diet could have contributed to less results, suggesting worse posture toward own health. If irregular access of antihypertensives, or uncorrected use by patients, the hypertension could not be controlled.

Non-treated hypertension suggests that alexithymia could be secondary to hypertension, explaining partially the misperception in this age interval (≤40). Depression and use of anti-hypertensive among A = D did not seem to interfere in weight perceiving. It might express levels of pro-inflammatory state. Depression itself can represent psychological elaboration of the own health state. The dimension of weight misperception in male who presented alexithymia, severe or moderate depression, and/or anxiety, was relevant in A > D, despite also present in female. Age and level of BMI (higher) seems linked with them.

A = D presented higher LDL (133 mg/dl), A > D higher total cholesterol (205 mg/dl) and hypertriglycerides (323 mg/dl). Neuro-inflammation could be linked with A > D.

### History of obesity, body mass index and weight perceptions

18% declared obesity during infancy in A = D, but 33% in A > D and 40% in A < D too; and obesity during adolescence: 33%, 52% and 48% respectively. 90% of A = D declared obesity in adulthood, more than A > D (73%). Obesity in A = D seems later acquired, in middle-age, than in A > D and A < D.

Differences statically significant (p ≤ 0.0001) were in types of weight perception considering levels of actual BMI: A < D prevalent in 79% of patients with BMII I and A > D in 68% of BMI III.

A > D could result of structural brain alteration in perception area, designed or not by cultural body size pattern. Such alteration seems opposite to misperception in anorexic patients: A > D patients probably did not perceive promptly their gain of weight, alterations in body size, despite changing due to plus weight. If linked with BMI III could be deleterious effect in brain, possibly related to difficulties in recognizing satiety such as compulsive behavior.

A = D, without weight perception alterations, was prevalent in BMI II. A < D associated to BMI I, seems a misperception did no related to structural brain alterations. Probably A < D, influenced by hypertension non treated, can suffer any temporary damage in such area.

### Psychological aspects and types of weight perception

Higher levels of depression were prevalent in patients who perceived A = D and A > D. Minimal levels of depression were prevalent in patients A < D (p = 0.049). Depression in both cases (A = D and A > D) can result of pro-inflammatory state of those patients, stressor events in their lives, or their awareness regarding their poor health. Since BMI I (minimal levels), but clearly ≥ BMI II, pro-inflammatory signs seems producing effects as depression.

35% of A < D accepted their physical appearance, contrarily of A = D (66%) and A > D (69%) (p = 0.0018). A < D seems genuinely a perception alteration, probably not as a result of a self-censure. 50% of A < D felt offended by social aggression about their weight; A = D (20%) and A > D (34%) (p = 0.007). The elevated percentage of patients who feeling target of social aggression in A < D, compared with the acceptation of their own appearance, could signalize denying (psychological mechanism): the censure remained “outside”, but denied by themselves. 69% of A < D was unsatisfied with themselves due to their disease (48% of A = D; 42% of A > D) (p = 0.027). As they were predominately BMI I and youngest, being portable of a disease could seem to them less acceptable. 74% of A < D felt unsatisfied with themselves because their obesity; A = D (51%) and A > D (54%) (p = 0.068). Despite A < D expressing highly unsatisfied with these items they were less affected by depression. The lesser was their dissatisfaction, in patients who presented A = D or A > D, the greater was their depression: as A = D could be psychological elaboration, but A > D probably linked with culpability, or damage in weight perception due to neuro-inflammation. Higher BMI and age, likely lesser the faith to achieve better results due to aging. Crescent the BMI, higher could be the conformism and lower the feeling to be able to fight against of obesity’s growing. A > D could be affected by emotional eating. They felt dissatisfaction with their obesity, more than A = D whose obesity seems recent than in A > D. BMI III if earlier linked with emotional eating, could after be intensified by cited brain damage.

A > D associated with BMI III, seems higher affected by pro-inflammatory effects. A > D linked with depression, BMI III and use of anti-hypertensive could be associated to irregular behavior regarding health, mainly males, who did not make diet, physical activity and treatments. If BMI III patients were greater emotionally immature their adherence to health habits could be compromised.

A = D was associated with BMI II and presented depression-related. This depression could be less associated with culpability, considering the accurate weight perception evolved. It can be related to a psychological elaboration process regarding their awareness of BMI, difficulties related to treatment, diet, aging and others aspects. It seems related to their health state and effect of their pro-inflammatory process which could reinforce levels of depression. Other sources of stress were not studied to explain prevalence of depression in BMI II. However, among female, climacteric and menopause can be linked with. They also used anti-hypertensive medication. It did not interfere in their weight perception. These patients could have regular attitude regarding the anti-hypertensive medication, following correctly instructions.

Patients who perceived A > D exhibited 3.6 more chances to present moderate or severe depression than A < D. A = D demonstrated 3.8 more chances to present moderate or severe depression than A < D. It is possible that in A = D, middle-age with their hormonal alterations, mainly amongst female, and initial stage of treatment’s against obesity due to recent acquisition of obesity, elevated the chances of depression-related to pro-inflammation. Gain of weight in middle-age could be culturally tolerated due to expectations about aging.

The higher the BMI, A > D was the prevalent type of perception. The lesser the BMI, A < D was the type; intermediate BMI, A = D. Despite A > D could be a type of perception reflecting structural brain alterations, it seems that pro-inflammation and depression-related were strongest associated to A > D such as bias in perceiving weight, reinforcing it as vicious circle. A < D, if not enough affected by pro-inflammation, presented a minimal depression. Since middle-age, and among BMI II and BMI III patients, depression was present in similar level.

Probable alexithymic patients were prevalent among A < D as a frontier (34%). It was present among in A > D (29%) and A = D (17%). Probable alexithymia and minimal levels of depression could be, beside non controlled hypertension, symptom of initial damages caused by obesity. The higher was the obesity expressed by BMI, the lesser was the presence of probable alexithymia. The distribution of probable alexithymia was opposite to the way to perceive weight: A < D (34%) and A > D (29%).

A < D, due to interval of age, could result of more rigorous expectations to deal with obesity and weight control, suggesting intern demand induced by external cues. A > D likely express, despite probable irregular use of anti-hypertensive and failed access to this medication, unstable attitude regarding treatment. A > D showed lesser patients earlier evolved in diet, during life, than A < D and A = D (p = 0.0091). It suggests worse attitude regarding eating, health and weight, lesser motivation or more negligence with health-items.

Patients who perceived their weight as A < D prevalently showed lower level of depression and less alexithymia (50%) compared with A > D and A = D (see Table 1). In A > D, alexithymia seems secondarily associated with depression.

Alexithymia, associated to BMI III and depression, seems susceptible to influences of brain alterations (amygdala, cingular gyrus and linked areas) already present. Imprecise perception of patients regarding items in general, including obesity, and different reaction in dealing with visual food stimulation can be linked with. Cognitive damages due to aging, low level of formal education and lack of information to behave correctly toward health, could still be associated.

Congruently the “pure” alexithymic (did not linked with depression or anxiety or both) was prevalent between A < D weight perception (43%). The Alexithymia associated with depression and/or anxiety was prevalent in those who perceived the weight A > D (22%). (see Table 1). The disapproval of their own appearance was present in those who perceived their weight as A > D (p = 0.0018) (a feature in male subjects). There was 2.5 more chances of alexithymia in A > D than A < D. Alexithymia was specially related to A > D, but A > D was strongly related to depression, mainly among BMI III. Their misperception is accompanied by lesser comprehension of their own feelings; part of them disapproved their appearance. If they did not be able to control factors as weight, it can signalize less adherence to treatments, which was indicated by males.

In A < D, the influence of depression was minimal, and the influence of alexithymia was lower than among other types of weight perception, if aged ≤40 years old. BMI I was prevalent between A < D. A < D was associated to hypertension, not treated by anti-hypertensive (p = 0.037**).** Alexithymia, if present amongst them, seems to be “pure” alexithymia (not associated with depression and anxiety). Whether there was less expressive effects of pro-inflammation, in A < D pro-inflammation, eventually neuro-inflammation, seems more relevant for alexithymia than depression.

Being ≤40 years old patients seems more relevant, beside civil state in lower level, reflecting developmental and cultural period of influence, with specific exigencies as: being “attractive” to achieve a partner, to obtain a stable profession and an occupational performance. If married the partner perception of weight can influencing self-image. Amongst non-married more stress, more lack of confidence in themselves and less skills to establish significant attachment could be evolved.

In A = D the differences in presence of obesity during infancy, adolescence and adulthood were more distinct: 6, 11 and 31% respectively. The differences of obesity at each period of development was less distinguishable in A > D (11, 18 and 25%), and in A < D (13, 15 and 27%). In A = D the presence of obesity since adulthood is higher than in other period of life-course. In A > D and A < D it was not greatly the difference between them in each period. A = D indicated less presence of obesity during infancy, while A > D and A < D were similar at this point.

In summary, 3 types of weight perception present distinguishable differences: A > D, A = D and A < D. A < D seems related to better self-control (BMI I) despite presence of obesity during infancy and adolescence. A > D seems reflecting lesser self controlled patients, with lesser diet during infancy and adolescence: possibly lack of model to better control their eating habits or lack of limits on food intake along life. A = D presented as main features: 3.8 more chances of depression than A < D (unhealthy state, beginning of treatment, probable elaboration of their disease and psychological state) and 4.3 more chances of alexithymia than A < D (62% of 41–60 year-old patients; many presented higher cholesterol, mainly LDL and hypertriglycerides; probably hormonal changes in female patients). A = D with alexithymia seems partially linked with higher cholesterol, indicating neuroinflammation due to hypertriglycerides. In middle age cognitive damages can initiate due to many factors: alexithymia could be expression of their unhealthy state. It did not induce patients to body image alteration.

The features of the patients who perceived A < D were: 89% did not use anti-hypertensive; 90% presented minimal depression; 50% were alexithymic and 34% presented probable alexithymia. Hypertension can influence alexithymia on A < D. The prevalence of probable alexithymia (38%) was in patients who did not use anti-hypertensive medication (uncontrolled hypertension). It can be observed prevalence (86%) of alexithymia among patients with controlled hypertension who used anti-hypertensive medication (p = 0.069). This difference, not statically significant, indicated an aspect in A > D: possible structural alteration.

A < D can be resultant of patients with greater aware regarding their actual health state: 74% declared to be unsatisfied with their weight (p = 0.068); 69% with their disease (p = 0.027). Youngest the patients, greater was their dissatisfaction with weight and disease.

A > D presented 3.6 more chances of moderate or severe depression (higher among males as already cited). Their depression here seems not accompanied by sentiments of fault, except in male who did not accept their appearance. Alexithymia, higher levels of depression (moderate or severe), higher levels of BMI (BMI III) seem importantly linked with A > D as a type of weight misperception.

Females, who declared had been anteriorly made diet as treatment to lose weight were exactly those who perceived their weight A > D (45%, p = 0.0091). Opposite attitude compared on male A > D.

Comparing on the patients who declared had been overweight during infancy one observed that A > D (33%) were different of those who perceived A = D (18%) (p = 0.073) and since adulthood (p = 0.009) period in which A = D was higher (90%) than A > D (73%), and A < D (86%). Even though not expressively, it can be observed that A = D and A < D were, in that case, more similar than A > D.

Higher was depression and alexithymia, crescent was the BMI. A > D seems expressing earlier beginning of obesity in life-course without diet, which explains more pro-inflammatory effects in psychological aspects and less diet during life. A < D express anterior starting of overweight than A = D, but both made treatments against obesity “earlier” (p = 0.0091).

If alexithymia is structural it partially explains less adherence to weight control and other items related to treatment. If associated to depression, it can be caused by pro-inflammation due to higher BMI (III) and their consequences. If it is an effect that did not contribute to the best comprehension of their won feelings and perception of weight, it can promote less adherence to diet and other treatments. If not structural, alexithymia seems an effect lack of model in general, beside lack of model to eating-control. It could also occur regarding feelings interpretation.

## Conclusions

Age, gender and civil state, in this sequence, presented descending order, in relevance, for weight perception. Males with alexithymia and severe or moderate depression and anxiety, but also females, presented fewer tendencies to correctly perceive weight. A = D presented lesser obesity during infancy and adolescence, explaining partially the lack of misperception between them.

Patients between 18 and 40 years old perceived with higher disapproving their weight, congruently with cultural and social demands of their life, but it seems with covert culpability among (or in) patients who “accepted” their appearance. Hypertension not treated could explain partially the alteration of perception achieved among them: A < D. The youngest the patient, the lesser was the actual BMI compared to the declared BMI; but younger was the patient, the greater their vulnerability to a pattern, contributing to this misperception.

It seems that the lower the acceptance of their own image the lower the admittance of reality, expressed by A > D, which seems present in patients with precocious beginning of overweight. A > D was linked with alexithymia, moderate or severe depression, BMI III and less diet anteriorly made during their lives if BMI III. Middle age or older were prevalent.

A < D and A > D were linked with higher presence of obesity during infancy and adolescence. A < D (BMI I) seems associated with better self-control than A > D (BMI III). A > D could be related to lack of model for self-control to eating, to interpret their feelings and body state, congruently with presence of alexithymia. Due to BMI III damages in satiety could be associated. Adolescence seems a crucial period in A > D.

Patients with Alexithymia who had A < D were less prevalent than patients with Alexithymia with A = D or A > D. Patients who perceived A = D seems realistic regarding weight due to their “recent” discovered of obesity. A < D, among youngest patients, presented more aware about their state, expressing higher dissatisfaction with their weight, appearance and disease. Their overweight began earlier in age than A = D, but not A > D. The higher was the BMI, the higher the levels of anxiety, depression and alexithymia in this sample.

One limitation of the present study is the size of the sample. Limits of the laboratories to present results sooner regarding cholesterol, LDL, triglycerides, HDL, glycemia and glycolysed hemoglobin, available in reports (1 month before, or later, the tests and questionnaire applied).

## Abbreviations

A = D: actual BMI = declared BMI
A > D: actual BMI > declared BMI
A < D: actual BMI < declared BMI
